# Family planning providers and contraceptive users in Rwanda employ strategies to prevent discontinuation

**DOI:** 10.1186/s12905-021-01503-1

**Published:** 2021-10-11

**Authors:** Hilary Schwandt, Angel Boulware, Julia Corey, Ana Herrera, Ethan Hudler, Claudette Imbabazi, Ilia King, Jessica Linus, Innocent Manzi, Madelyn Merritt, Lyn Mezier, Abigail Miller, Haley Morris, Dieudonne Musemakweli, Uwase Musekura, Divine Mutuyimana, Chimene Ntakarutimana, Nirali Patel, Adriana Scanteianu, Biganette-Evidente Shemeza, Gi’anna Sterling-Donaldson, Chantal Umutoni, Lyse Uwera, Madeleine Zeiler, Seth Feinberg

**Affiliations:** 1 Western Washington University, 516 High Street MS9118, Bellingham, WA 98225 USA; 2 Spelman College, Atlanta, USA; 3 Wheaton College, Norton, USA; 4 Northwest Vista Community College, San Antonio, USA; 5 Whatcom Community College, Bellingham, USA; 6 INES, Ruhengeri, Rwanda; 7 Xavier University, New Orleans, USA; 8 University of Maryland-Baltimore County, Baltimore, USA; 9 SUNY Oswego, Oswego, USA; 10 Western Oregon University, Monmouth, USA; 11 Eastern Oregon University, La Grande, USA; 12 University of Kentucky, Lexington, USA; 13 Arcadia University, Glenside, USA; 14 Rutgers University, Newark, USA; 15 Drexel University, Philadelphia, USA

**Keywords:** Contraception, Strategies to prevent discontinuation, Switching, Rwanda

## Abstract

**Background:**

In Rwanda, nearly a third of contraceptive users discontinue within the first year of use. Family planning programs often focus more on recruitment of new users as opposed to maintaining use among current users. A focus on sustaining users and minimizing discontinuation is imperative for long-term family planning program success. This study explores the efforts providers and contraceptive users in Rwanda employ to prevent one of the greatest challenges to family planning programs: contraceptive discontinuation.

**Methods:**

This was a qualitative study conducted in Rwanda between February and July 2018. It included eight focus group discussions with 88 family planning providers and 32 in-depth interviews with experienced modern contraceptive users. Data were collected in two districts with the highest (Musanze) and lowest (Nyamasheke) rates of contraceptive use. Data were analyzed using thematic content approach.

**Results:**

Family planning providers in this study used the following strategies to prevent discontinuation: counseling new users on the potential for side effects and switching, reminding clients about appointments for resupply, as well as supporting dissatisfied users by providing counseling, medicine for side effects, and discussing options for switching methods. Users, on the other hand, employed the following strategies to prevent discontinuation: having an understanding that experiences of side effects vary by individuals, supporting peers to sustain use, persisting with use despite experiences of side effects, and switching methods.

**Conclusions:**

The strategies used by family planning providers and users in Rwanda to prevent discontinuation suggest the possibility of long-term sustained use of contraception in the country. Harnessing and supporting such strategies could contribute to sustaining or improving further contraceptive use in the country.

## Introduction

Approximately 215 million women in the world wish to avoid or delay pregnancy yet do not use family planning methods [1]. If family planning needs were fully met, there would be a 67% reduction in unintended pregnancies, 70% fewer maternal deaths, 44% fewer newborn deaths, and 73% fewer unsafe abortions [1].

Discontinuation of contraceptive methods, despite a desire to continue to avoid pregnancy is very common and a major challenge for family planning programs. Due to contraceptive discontinuation differing by method type, discontinuation is examined among and disaggregated by many types of contraceptive methods, such as natural family planning methods (LAM, standard days, withdrawal, and rhythm), male condoms, and reversible hormonal methods (pills, injectables, implants, and IUD).

Researchers examining data from 36 developing countries estimated that approximately a third of unintended births are due to contraceptive discontinuation [2]. Globally, these findings suggest that 25 million of the 75 million unintended births are due to contraceptive discontinuation [2]. Studies show discontinuation rates between 6 and 56% after just 12 months of contraceptive use [3–14]. Discontinuation, while still in need of pregnancy protection, increases with time beyond the most common measure of after 12 months of use [3, 9, 13]. Discontinuation has been reported as significantly more common in sub-Saharan Africa compared to other world regions [15]. Often the most common reasons for discontinuation for those who wish to continue to prevent pregnancy is method related—such as health concerns or the experience of side effects [3, 7, 9, 12, 16]. Other less common reasons for discontinuation, while still wanting to prevent pregnancy, include switching, a desire for a more effective method, and method failure [17]. Research in Uganda examining the impact of consequences of contraceptive use on discontinuation and switching found that heavy or prolonged bleeding increases the risk of discontinuation while reduced libido contributes to increased switching behavior [18].

Switching from one method to another is the ideal course of action for women and couples who desire to continue to prevent pregnancy but are not satisfied with their current method. A study in 23 low and middle income countries found switching to be around 60% within three months [16]. Researchers examining switching after one year of use found rates between 10 and 80% [3, 7, 9, 12, 14]. Researchers have found lower levels of switching in sub-Saharan Africa countries than in other regions of the world [3]. A study in Kenya found that 63% of discontinuers had switched to another method within three months of discontinuation, while another study in Malawi found switching within three months of discontinuation was just 4% [11].

In Uganda, a nation characterized by low modern method use, high rates of discontinuation, and high fertility, a qualitative study on contraceptive use experience found that women were motivated to sustain contraceptive use despite side effects, peer support varied by contraceptive experience, and that healthcare providers played important roles in women’s contraceptive use decisions. Women were motivated to use contraception despite experiencing side effects due to the need to ensure healthy growth of their existing children. Peers who had positive contraceptive use experiences supported their friends in sustaining the use of their methods while those with no or negative experiences discouraged their friends from using contraception. Health providers supported women experiencing side effects by providing counseling, medication to relieve side effects, as well as support with switching methods [19]. Literature suggests various strategies that providers should use to prevent discontinuation, including proactively counseling users on potential side effects, reminding users to return for resupply, assisting users who return due to unmanageable side effects, and supporting users in switching methods [2, 3, 10, 11, 20].

Examining experiences of contraceptive discontinuation and strategies to prevent it from the perspectives of users and providers is important, especially in contexts where the desire for large family sizes is declining and contraceptive use is increasing. This profile fits the nation of Rwanda—where the mean ideal family size among all women declined from 4.3 to 3.3 between 2005 and 2010 [17]. During the same period, modern contraceptive use among currently married women increased from 10 to 45% [17].

Rwanda is also characterized by high rates of adult literacy (80%) [17] and has a culture of monthly community workdays (*Umuganda)* that are mandatory for all citizens, which often involves government-sponsored messaging on the importance of family planning. Gender equity has also been a focal point of Rwandan governance as the nation rebuilt itself from the civil war and genocide that ravaged the population between 1990 and 1994 [21]. While patriarchal foundations are still ubiquitous in Rwanda, women play prominent roles in community organizations at the local level and make up a higher representation in the national legislature (61%) than any other country [22].

Rwanda’s family planning program has been recognized as successful due to the rise in the use of modern contraceptives in the country [23–25]. Injectables comprise the largest share (24%) of the method mix in Rwanda, followed by pills and implants (8% each) [17]. Estimates from the 2014–2015 Rwanda Demographic and Health Survey show that 28% of family planning users in the country discontinued a method within 12 months of initiating use—with 34% discontinuing due to health concerns and side effects—the most common reason for discontinuation [17]. Discontinuation was lowest for implants (3%) and highest for pills (42%) [17]. Ten percent of users switched methods, with 21% of pill and 15% of male condom users switching methods, compared to less than 1% of implant users [17]. This paper examines strategies family planning providers and users employ to prevent discontinuation in the context of increasing use of modern methods, low rates of discontinuation, and method switching.

## Methods

### Study setting

This qualitative study took place in Rwanda in 2018. Data collection activities took place in the Musanze district in northern Rwanda and the western Nyamasheke district. Both districts have similar population sizes of around 375,000 inhabitants; however, the population in Musanze is more urban (28%) than Nyamasheke (2%) [17]. In addition, the proportion of women with secondary or higher levels of education is higher in Musanze than in Nyamasheke (19% and 14%, respectively) [17]. The total fertility rate in Nyamasheke is 5.0 children per woman while it is much lower in Musanze at 3.5 [17]. These two districts were purposively selected based on Demographic and Health Survey data showing that Musanze had the highest modern contraceptive prevalence rate while Nyamasheke had the lowest [17].

### Study design and sample

This cross-sectional study included both focus group discussions and in-depth interviews. Eight focus group discussions, ranging in size from 8 to 12 participants, were held in February of 2018 with 88 family planning providers. Focus group discussions were utilized to better understand the group norms among family planning providers. Each lasted two hours on average. Four of the focus group discussions were conducted with family planning nurses (2 in each district), who worked at hospitals, health centers, or health posts, and the other four were held with community health workers (CHWs) (2 in each district). CHWs are locally elected community members who are trained by the Rwandan government and tasked with providing family planning information to community members, referrals to the nearest health facility, and condoms as well as resupply of pills and injectables. The nurses and CHWs were selected to capture the experiences of both institution and community-based providers.

Thirty-two in-depth interviews were conducted in July of 2018 with mostly current modern contraceptive users who were at least 18 years old. The interviews lasted on average 43 min. The interviews captured the personal experiences of participants with contraceptive use and discontinuation.

Staff from a local non-governmental organization (NGO) that worked with family planning providers as well as those from government hospitals who supervise family planning providers at the district level assisted with recruitment of nurses and CHWs. They recruited participants by reaching out to them to tell them about the study, provide details, and gauge interest. The providers who participated in the study then assisted with the recruitment of experienced contraceptive users through phone or in person conversations, provided them with details about the study, and gauged their interest in participating in the research.

The initial target was to recruit 16 current users of modern contraception who had been using a method for at least six months and 16 who had discontinued a modern method within the last six months, for reasons other than desiring a pregnancy. The goal of using the cut-off of at least six months was to avoid interviewing inexperienced contraceptive users who had just initiated or discontinued use and did not have much experience to draw from for the interviews. During recruitment of contraceptive users, it was apparent that the study definition of discontinuation was not clear—as women who had discontinued a hormonal method and then switched to condoms were classified as discontinuers by the recruiters. Finding women who met the study definition of discontinuation was a challenge. By the end of the study, 13 “discontinuers” were included but of these, six were currently using condoms.

### Data collection and analysis

The focus group discussions were each led by a moderator who was assisted by a note-taker and took place inside private rooms at government buildings identified by the local study collaborators. The in-depth interviews were each conducted by one interviewer and took place inside each participant’s home, or outside, based on the study participant’s choice and the ability to find private space for the interview. All the data collectors were fluent in Kinyarwanda—the first language of all study participants, and all data collection activities were conducted in Kinyarwanda. The data collectors completed an extensive training prior to data collection—that included review of study aims, research ethics, as well as extensive review and practice using the topic guides. Data collectors were trained to use the questions as a guide—not a survey, and to allow the conversations to flow naturally. The topic guides remained unchanged after pilot testing with the data collectors. The data collection activities were audio-recorded with the consent of participants.

Translation of interviews from Kinyarwanda to English occurred during the transcription process by the data collectors, who were native Kinyarwandan speakers also fluent in English, in collaboration with native English speakers. The English transcripts were then transferred into Atlas.ti 8 [26] for coding. Thematic content analysis, which involved finding repeated themes of meaning across all transcripts, guided data analysis [27]. Two teams of researchers each coded the same first two transcripts—and then collaboratively created the universal codebook. The teams then used the codebook to code the remaining transcripts. The codes were exported to Excel and analyzed by examining relationships between and within codes to generate themes and sub-themes. Data from focus group discussions with providers were triangulated with data from interviews with contraceptive users.

### Ethics

Ethical approval to conduct this study was obtained from the Institutional Review Boards at Western Washington University and the Ministry of Education of Rwanda. Participation in this study was completely voluntary. Every study participant read and signed a consent form prior to participation in the study. Study participants each received 10,000 Rwandan francs (approximately $11 USD) to cover transportation to and from the data collection venue.

## Results

### Socio-demographic characteristics of participants

Among the 88 family planning providers, 52% were CHWs and 73% were female. The average age was 44 years, ranging between 28 and 61 years. Providers had been working on average 6 years, ranging between 1 and 22 years. Among the experienced contraceptive users, 91% were married. The average age of the women was 38 years, ranging between 26 and 50 years. They had four children on average, ranging between one and nine children. Most participants used injectable (41%), followed by condom (22%), implants (19%), pill (12%), while 6% were sterilized. On average, participants used their current method for 6 years, ranging between 6 months and 25 years.

### Topics

The results fall into four overarching topics: side effects as reasons for discontinuation, how discontinuation is realized, efforts to prevent discontinuation, and the outcomes of efforts to prevent discontinuation.

## Side effects as reasons for discontinuation

In the overarching topic of side effects as reasons for discontinuation there are two subthemes: fears of side effects and experiences of side effects.

### Fears of side effects

When discussing fears related to contraceptive use, the main theme that arose among study participants was that of side effects. Women experienced fear of side effects prior to initiating contraception.: “I have tried to fight against the feelings that using family planning will put me at risk” (Female, pills for 4 years, 32 years old, 3 children, limiter, Musanze). Women from Nyamasheke expressed fear of side effects more frequently than those from Musanze.

Some current users still feared that side effects could occur in the future.

- I: Did you have any fears about the side effects of using this method?
- R: Yes, and I still have that fear. I think the fears will stop after the three years because I am still nervous about the side effects.
  - Female, implant for 6 months, 29 years old, 2 children, spacer, Nyamasheke

For others, the experience of side effects created fear about potential future experiences of the same, which impacted method choice.

- I will not try another method because I am afraid of having other effects because the ones I tried didn’t work. I will continue to use condoms.
  - Female, condoms for 10 years, 40 years old, 4 children, limiter, Nyamasheke

Some participants reported that rumors about contraceptives induced fear in them, or in others, and thus affected their readiness to use contraception.

- I knew many rumors that said kids will grow funny if you use family planning and it will cause many side effects, so I refused to use it.
  - Female, implant for 6 months, 29 years old, 2 children, spacer, Nyamasheke

Contraceptive users in this study, more so among users in Nyamasheke than in Musanze, reported that fears of side effects impacted their contraceptive use decisions in terms of delaying initiation of use, remaining fearful during use, and method choice. Fears of contraceptive side effects originated from experience or rumors.

### Experiences of side effects

Experienced contraceptive users mentioned the following side effects, in order of frequency: excessive or prolonged menstruation, dizziness, headache, amenorrhea, fatigue, weight gain, loss of libido, and weight loss. Contraceptive users from Nyamasheke reported experiencing side effects during interviews more frequently than those from Musanze.

- The experience I have gotten from the methods I was using was not good. From the first method (injectable for three months) I missed my period, I gained too much weight and I got back pain. Then from the implant I was bleeding—when I was having my period one week, after one week again I would have my period, so like 15 days in a month. The implant was the worst compared to the injectable. After that, the condoms I am using don’t cause too much trouble with my health. I am getting my period regularly for three days or four and then it’s gone. And also, my weight has reduced.
  - Female, condoms for 10 years, 40 years old, 4 children, limiter, Nyamasheke

Some women complained of side effects without being specific while others noted serious side effects not typically associated with hormonal contraceptive use, such as noted by the injectable user below.

- …when I used the injection, I suffered from back pain and shivers in my body.
  - Female, former injectable user, 32 years, 3 children, spacer, Musanze

Another theme that emerged from the narratives was the absence of side effects. Reports of absence of side effects were nearly three times more common among participants from Musanze than Nyamasheke.

- The experience I had in using the injectable for 10 years is that I never had any side effects or any problem with that.
  - Female, implant user for 4 years, 35 years old, 4 children, limiter, Nyamasheke
- I: How much longer do you plan on using this method?
- R: I cannot say the exact time that I want to use this method because it is currently working well for my body and I think that I could continue using this method, unless there are any side effects that may come up, but other than that I will continue using it forever.
  - Female, injectable user for 6 years, 32 years old, 2 children, spacer, Nyamasheke

Most participants reported absence of side effects as an aspect of their entire experience but presented it as their main experience. Many women tried multiple methods before they found a method that worked for them—and sometimes a method that worked previously no longer worked after a break or after a period of sustained use.

- After three years of using the injectable, I started seeing changes, like during my menstruation period I started to get dizzy and fall down.
  - Female, condoms, 37 years old, 4 children, limiter, Nyamasheke

Sometimes women reported experiencing serious side effects, and having no side effects, in the same interview. This occurred even when reporting about experiences using the same method—as demonstrated by this participant about her experiences with injectables early in the interview:

- I was bleeding so much and I lost weight and I was about to faint.

Then later in the interview, she reported:

- Until now, I haven’t had any problems or side effects, and I don’t think that I will have any problems in the future. Every time I go to see the doctor to check if everything is okay, it is.
  - Female, injectables for 10 years, 50 years old, 5 children, limiter, Musanze

Discussion about contraceptive side effects featured in every interview although the emphasis varied by study setting. Among Nyamasheke participants, there was more emphasis on the details of specific side effects while among Musanze participants the emphasis was more on the absence of side effects. In addition, some participants emphasized absence of side effects despite reporting experiencing the same in other sections of the interview while others reported complete absence of side effects.

## How discontinuation is realized

In the overarching topic of how discontinuation is realized there are two subthemes: taking temporary breaks and switching from hormonal methods.

### Taking temporary breaks

Participants reported that the main reason for recent, or past, discontinuation of contraceptive methods was side effects.

- I stopped using family planning in order to give my body a break, and I plan to continue using family planning after that break…
  - Female, traditional methods, 34 years old, 3 children, spacer, Musanze

Discontinuation of contraception was always considered a temporary break due to unmanageable side effects—participants were keen to resume contraception to achieve their child spacing and limiting goals.

### Switching from hormonal methods

Contraceptive users stated they discontinued—but they often switched from a hormonal method to a non-hormonal method—either condoms or traditional methods. Study participants viewed only hormonal methods as contraceptive methods.

- I stopped using them because I had headaches and there were times when I felt like I couldn’t see clearly. For now, I am using condoms, but because I had those headache and vision problems, I stopped the implant. I stopped because I had those side effects, but when the problems subside, I will use another type of contraception.
  - Female, condom user for 2 years, 35 years old, 4 children, limiter, Nyamasheke

Participants reported that health care providers tried to help alleviate the side effects, and when that did not work, advised the users to pick a method with no side effects in between discontinuing their current hormonal method and choosing a different hormonal method.

- I: Why did you discontinue using pills?
  R: The pills caused me to have more stress, headaches, amnesia, and bleeding. Then I decided to discontinue, and I went to the health center and after testing me they told me that I had to use condoms or the calendar method while I thought about another method I could use.
  - Female, condom user for 6 years, 38 years old, 4 children, limiter, Nyamasheke
- Before I discontinued, I went to the health center and they gave me some drugs because I was having more bleeding and high blood pressure with the five-year implant, but there was no change. … And then, the nurses and doctors helped me take out the implant. They advised me to go and take some rest and to use condoms and the calendar method while I thought about the method that I may use next.
  - Female, former implant user, 38 years, 5 children, limiter, Nyamasheke

Contraceptive users in this study described discontinuation as switching from a hormonal method to a non-hormonal method, most often condoms and/or a traditional method. Discontinuation occurred due to unmanageable side effects and was considered temporary to allow side effects to subside. Providers made efforts to help users manage side effects, and if those efforts did not work, encouraged women to take temporary breaks from hormonal methods while using non-hormonal methods. Both contraceptive users and providers were keen on women resuming hormonal contraception. Implied in their discussion was the preference for hormonal methods because of their effectiveness compared to short-term non-hormonal methods.

## Efforts to prevent discontinuation

In the overarching topic of efforts to prevent discontinuation there are six subtopics: providers help clients prepare for side effects and switching, providers remind clients about appointments for resupply, understanding side effects, sustaining current method use, providers counsel women who want to discontinue, and women advise others to persist with use despite side effects.

### Providers help prepare clients for side effects and switching

Providers and contraceptive users both spoke about the importance of counseling clients about potential side effects prior to use.

- Before I started using family planning, they told us about all methods, and they told us about the impact we can have from those methods. The injectable was the choice I made. After, I had side effects from the injectable, but I knew it would happen because they told us about it.
  - Female, condoms for 5 years, 40 years old, 5 children, limiter, Nyamasheke
- R: From the day I started the injectable I didn’t get my period.
  I: Did you have a problem not having your period?
  R: I had no problem with that because the nurses and doctors told us to expect that.
  - Female, condoms for 5 years, 40 years old, 5 children, limiter, Nyamasheke

Family planning users often reported that providers also assisted new adopters during initial counseling to prepare for switching if side effects became unbearable.

- …the community health workers told us when one method becomes too difficult to continue using you can shift (*switch*) methods.
  - Female, sterilized, 43 years old, 5 children, limiter, Nyamasheke

Family planning providers and users reported that providers counsel users on their method choice, including discussions about possible side effects as well as the potential need to switch to a different method based on the side effect experience. Women’s reports of side effect experience were influenced by initial counseling about potential side effects. In particular, information provided in advance made the women’s experience of side effects bearable.

### Providers remind clients about appointments for resupply

Family planning providers informed new users about the potential for side effects and the possibility of switching—they also helped facilitate continued use among users via reminders about upcoming appointments. Contraceptive users reported that the family planning providers gave them appointments to return for resupply of their contraceptive method. Some contraceptive users reported that providers, particularly CHWs, reminded them, most often with a phone call, if they missed their appointments.

- …when that appointment comes, they (providers) reach us so we cannot miss our method.
  - Female, condom user, 38 years old, 2 children, spacer, Nyamasheke

CHWs were motivated to help women honor their appointments to avoid issues of unplanned pregnancies or the need for testing for pregnancy if too much time lapsed between contraceptive coverage. Contraceptive users were similarly motivated to keep appointments to avoid unplanned pregnancies.

- They gave us the date to come to take the injectable, so we try not to miss that date in order to avoid unplanned pregnancy. And ourselves, we try to respect that day because when you miss that day, you get many challenges in your life.
  - Female, injectable for 7 years, 31 years old, 4 children, spacer, Musanze

Providers and contraceptive users reported that providers ensured that they reminded women about appointments for resupply. The motivation for providers and users to ensure women kept appointments was pregnancy prevention—there was no mention of the potential for mitigation of side effects with timely resupply.

### Understanding of side effects

Study participants reported that people’s bodies differ—so one person’s challenges with contraceptives may not necessarily be the experience of others. Women noted this as general advice—and also spoke about their personal experiences.

- …every woman responds differently to family planning methods.
  - Female, former implant user, 38 years, 5 children, limiter, Nyamasheke
- She has to talk to the doctor about which method she should use according to her unique body. There are women who use contraceptives who never have problems the whole time, and there are some women who have side effects after using contraceptives for a month.
  - Female, condom user for 2 years, 35 years old, 4 children, limiter, Nyamasheke

There was an awareness that hormonal methods’ interference with the body most likely occurs at the beginning of method use; however, some women reported that such interference can change after using a method for a long period of time.

- By now, I can’t predict the time for how much longer I will be using this method because now it has to do with my body, but maybe I may change after ten years when I see… when my body stops fitting to the method I am using (*side effects begin*).
  - Female, pills for 4 years, 32 years old, 3 children, limiter, Musanze

Contraceptive users indicated that bodies differ; therefore, some people might have many challenges with contraceptive use, while others may have just a few, or even none. Additionally, there was an awareness that challenges are most likely to occur during initiation of use—but also that challenges could arise at any time during use. Women were of the view that this should encourage others to use contraception while being prepared for side effects if they arise, and have switching plans if side effects become unbearable.

### Sustaining current method use

Women were reluctant to switch methods if they were not experiencing side effects. Half of the sample used only one method for, on average, seven years—most often injectables. Sustained use of the same method for long durations was more common in Musanze than in Nyamasheke.

- I don’t want to switch because I have never had side effects so I do not want to take the risk, maybe if I switch, the other method I will try will be bad.
  - Female, injectable user for 10 years, 38 years old, 5 children, spacer, Musanze
- I will always use this method of pills, maybe forever …I don’t have plans to change or to switch to another method because these pills work well for me. If these pills work for me why would I change to another method? I will stay with this one.
  - Female, pill user for 3 months, 34 years old, 2 children, spacer, Musanze

Some women reported a fear that if they switched methods, challenges would arise with the new method, which could then lead to discontinuation. They therefore preferred to continue using the same method to avoid any challenges that might result in discontinuation. Such fear led some participants to use short-term methods for long periods.

### Providers counsel women who want to discontinue

Providers and contraceptive users reported that women should return to the clinic when they experience unmanageable side effects as opposed to turning to others in the community for support due to concerns that social networks would respond by sharing myths and rumors about contraceptives as opposed to supporting sustained use. The advice to return to the clinic was made more often by nurses than CHWs.

- The advice I would give her is that she should not ignore it. I would tell her that if she has those side effects, she should go to see a nurse who will try to find a way to help her.
  - Female, condoms, 37 years old, 4 children, limiter, Nyamasheke
- If your body is not responding well, you have to go to the health center to get help because if she doesn’t go to a doctor, she goes to the neighbors or other women, is where she will get rumors that the contraceptive method is not good.
  - Nurse, female, 50 years old, 5 children, sterilized, Nyamasheke

Providers indicated that when women choose to use contraception to either limit or space their births, then the only response to this desire was to find a method that worked to assist the client in achieving this goal.

- If someone wants to stop using family planning, if they come to me as a CHW I will show them first the world they live in and how small it actually is. And then I will show her how difficult it is to find her child milk…. I will let her make the decision of using family planning or not. In short, I will say teaching her so that she might not be disappointed in stopping and I will give her examples by showing her her neighbors and friends and after that she might have courage in family planning.
  - CHW, male, 56 years old, 5 children, implant user, Musanze

Family planning providers reported that they asked clients about their plans to discontinue and their reasons so they could properly counsel them on all their options. Providers sought to assist women in continuing to use contraception when the women desired to prevent pregnancy. It takes time for family planning providers to counsel dissatisfied users—to help them find solutions with their current methods or to educate clients about alternative methods. Providers did not explicitly state this time burden, or identify it as a burden, but their comments suggested that efforts to help dissatisfied users can take significant time.

- After understanding how all the different contraception methods can be used, she can then find one more suitable if she chooses to quit using the first one.
  - CHW, female, 51 years old, 5 children, injectable user, Nyamasheke
- If I understand why she stopped using the contraceptive method, I can start over by teaching her again about contraceptives and see if I can change her mind from the decision she made to stop using the contraceptive method.
  - Nurse, female, 2 children, non-user, Musanze
- As she goes to the CHW to change the method, the CHW will not tell her this is the best method for you. They will explain to her again about other methods and she will choose a method on her own that she wants to use.
  - Nurse, female, 2 children, implant user, Musanze
- What the CHW should do is give her time to explain and pay attention to what she is saying. Maybe she should come when there are less clients based on the appointment she is given so that the CHW has enough time to meet with her. It will allow her enough time to feel free to express those challenges and be given another method.
  - CHW, female, 48 years old, 6 children, condom user, Nyamasheke

A few providers reported that they would counsel clients on the potential consequences of discontinuation when still desiring to avoid an unwanted pregnancy.

- I would advise them about the challenges they can face if they stop using family planning. When you don’t use family planning you can have too many kids to care for based on your financial circumstance, they may face malnutrition.
  - CHW, female, 45 years old, 6 children, injectable user, Nyamasheke

Both contraceptive users and providers discussed the importance of returning to providers when challenges with use arise. Both groups of participants preferred that women receive support from providers and not community members to continue use or switch methods. Women noted that turning to social networks with complaints regarding contraceptive use would likely result in encouragement to discontinue due to rumors about method use.

### Women advise others to persist with use despite side effects

Study participants reported that they often advise their friends and neighbors to stick to using family planning despite the issues that arise with using hormonal contraceptives—that the side effects would pass with time so they should persist in using the method of family planning they were currently using.

- …I tell them don’t be discouraged, continue using family planning.
  - Female, injectable for 25 years, 50 years old, 8 children, limiter, Musanze
- If you find that none of those methods works for you, you can ask a nurse for advice, but you cannot start using contraceptives for two months and then say that you are going to stop.
  - Female, condoms, 37 years old, 4 children, limiter, Nyamasheke

Women reported that they would encourage others to recognize that individuals react differently to different methods, and that one should persist in finding the right method that suits her.

- For me, when I used injections I did not have good luck with them, but I would say to them that they might be good for you, and if using injections doesn’t work for you, then you can use pills.
  - Female, pill user for 10 years, 38 years old, 3 children, limiter, Musanze
- Another piece of advice for someone who has side effects is that she doesn’t have to stop completely for fear that if she uses another method it will cause problems. I think she should try and try until she gets the type of contraceptive that fits her body (*suits her*).
  - Female, pill user for 3 years, 45 years old, 2 children, spacer, Nyamasheke

Women reported that they supported others to continue using contraception through encouragement, sharing awareness that side effects can lessen with time, and suggesting trying and switching methods until the user finds the right method for them.

## Outcomes of efforts to prevent discontinuation

In the overarching topic of outcomes of efforts to prevent discontinuation there are two subthemes: switching between hormonal methods and persistence of use despite experiencing side effects.

### Switching between hormonal methods

The topic of switching between methods was mentioned more frequently by study participants than discontinuation. Providers and women reported that the most common reason for switching was unmanageable side effects.

- First, I was using the three-month injectable, and I only used it for three months and then I had side effects. I found that this method didn’t work for me well and I switched to pills, and I am using pills now.
  - Female, pill user for 3 years, 45 years old, 2 children, spacer, Nyamasheke

Providers often responded to complaints about unmanageable side effects with a desire to counsel the client on other options and to assist them in identifying a new method. Providers rarely mentioned the option to discontinue a method.

- Due to the side effects she has when using the method, it will be difficult for nurses to convince her to continue with that method. That’s why I said she will leave with another method.
  - CHW, male, 54 years old, 5 children, non-user, Nyamasheke
- I tried to talk to a doctor, and asked them, “Since injections gave me lots of problems, what else can you offer?”…The nurses gave me advice to switch methods and go to the pills, and they said that if the pills didn’t work for me that I can come back and see what else I can use.
  - Female, pill user for 10 years, 38 years old, 3 children, limiter, Musanze

Providers reported they often worked with clients to continue using their current method despite experiencing side effects and offered switching methods as an alternative if their efforts to mitigate side effects failed. Again, discontinuation was not offered as option under such circumstances.

- Just because she experiences a side effect does not mean we must change the method of which we have used. As providers we have ways to treat various side effects, so that she may continue to use that method. However, if it is not treatable, we will consider other contraceptive methods.
  - Nurse, female, 36 years old, 3 children, implant user, Musanze

For some current users interviewed, the number of methods the women had tried, and switched to, was high. While two women had tried four methods, the average number of methods the women had used was two.

Providers and women reported that women sometimes switched from short-term methods to long-term methods—less so for increased effectiveness but rather for their long-term nature—particularly to limit the need to visit a health provider at regular intervals to free up time to engage in economic opportunities.

- The reason why she might want to stop the pill is because all the time she was going back and forth to the hospital, she could be doing something for her family. Instead of being in the same situation, she chose to use a longer method.
  - CHW, female, 50 years old, 2 children, non-user, Musanze

Providers were motivated to help women sustain use—and through their efforts one can see their preference for women to continue using their current method, as opposed to switching, likely due to an awareness that side effects are more common when initiating use as opposed to during sustained use. However, providers were keen to recommend switching when women were unwilling to sustain use, and rarely, if ever, recommended discontinuation.

### Persistence of use despite side effects

Over half of respondents expressed a desire to continue using family planning despite experiencing side effects.

- I went to him (CHW) and tried to discuss my problems with him, and I tried to show him the difficulties I have with using pills…but also that I didn’t want to stop using them.
  - Female, pill user for 10 years, 38 years old, 3 children, limiter, Musanze
- I was bleeding so much, and I lost weight and I was about to faint. The doctor told me to go home and continue using the withdrawal method. That is when I refused to go home, and I insisted that they give me the injectable again.
  - Female, injectables for 10 years, 50 years old, 5 children, limiter, Musanze

Respondents reported that finding ways to deal with the side effects of contraceptives was much better than the alternative of not using family planning.

- … even though I experienced side effects when I started using family planning, if I had given up using family planning in the first place I would have more children so I continued to use it even though I knew that I was passing through a hard time with the side effects until we had the children we wanted to have.
  - Female, condoms for 10 years, 40 years old, 4 children, limiter, Nyamasheke
- …using injectables helped me to not get pregnant when I didn’t want to get pregnant. I experienced side effects but the injectable helped me to not get pregnant.
  - Female, pill user for 3 years, 45 years old, 2 children, spacer, Nyamasheke

Women’s sustained use of contraception, despite side effects, demonstrates their motivation to achieve their desired child spacing and limiting goals and their awareness of the greater burden of an unwanted pregnancy compared to side effects of contraception.

## Discussion

The findings of this study show that discontinuation was rarely an option for users experiencing side effects. Once a couple decides to use contraception to achieve their birth spacing or limiting goals, their providers aim to help them achieve those goals through supporting sustained use or recommending switching methods, despite experiencing side effects. This is consistent with findings from a study in Uganda that found that contraceptive users were determined to continue use despite side effects [19]. Family planning programs can design behavior change communication to encourage a focus on meeting child spacing and limiting goals, including persisting with use despite side effects. In tandem, programs can train providers to support users in their efforts to meet their child spacing or limiting goals.

As with contraceptive users worldwide, participants in this study were most fearful of side effects when contemplating use of contraceptives—and even during use [4, 19, 28–30]. Although many contraceptive users reported experiencing side effects, some expressed a desire to downplay such experiences and present their overall experience as positive. Family planning programs can encourage potential clients to try contraception through messaging that highlights users who have experienced side effects and successfully managed those side effects.

Contraceptive users and family planning providers alike reported that providers counseled new users about the likely experience of side effects—as well as the possibility of switching if side effects became unbearable. Many researchers found that provider counseling on side effects and switching reduced contraceptive discontinuation [2–4, 6, 7, 10, 11, 15, 19, 28–33], while others suggest that programs and providers have little impact on continuation [13, 34]. One study analyzed the correlation between discontinuation rates and family planning program effort scores, and found a negative relationship, albeit weak, and concluded that family planning programs can impact discontinuation, particularly in terms of training and supervision of providers [5]. Another study tested a client-centered approach to family planning counseling and found that the intervention was successful in increasing provider, as well as client, knowledge of methods and side effects [35]. Others found lower discontinuation rates among clients who felt they were adequately counseled compared to other clients who felt they were not adequately counseled [31, 32, 36, 37]. The findings suggest a need for family planning programs to train and support providers to proactively discuss side effects with new and continuing users and to offer options for switching in cases of unbearable side effects.

Switching of contraceptive methods was common among some of the study participants. Participants also reported taking “temporary breaks” from hormonal methods. These breaks were described as a break from using family planning methods, even though they often entailed switching to less effective methods. A similar finding emerged from research in Ghana where study participants did not consider less effective methods family planning methods [29]. The findings suggest the importance for research on contraceptive discontinuation to discern women’s understanding of the term for more nuanced measurement of actual levels and patterns of discontinuation.

Both contraceptive users and providers advised women experiencing side effects against turning to their friends and community networks for assistance due to concerns that social networks would respond to contraceptive complaints by sharing rumors about contraceptives, thereby discouraging continued use. Researchers have shown the negative impact of myths on sustained contraceptive use [4, 19, 28, 38, 39]. Myths about contraceptive use were nearly absent from both the focus group discussions and the in-depth interviews—the emphasis lied more in avoiding myths and rumors than in actual discussion of what the myths were. Instead, the advice was to return to the professionals for help, as providers would support sustained use. By returning to providers for assistance, the study participants believed that women would be less likely to be negatively affected by myths and rumors about contraceptives—and by extension, would remain more successful in continuing to use contraception. Research in the Democratic Republic of Congo has demonstrated the positive impact of clients returning to providers for assistance with side effects instead of community members [7]. Family planning programs can support sustained use of contraception by supporting providers in welcoming back unsatisfied users and establishing community norms about the importance of returning to providers when experiencing side effects. Additionally, family planning programs could tap into experienced consistent contraceptive users as champions for addressing myths and misconceptions in their communities.

Contraceptive users who did not experience side effects were reluctant to change methods as they feared switching methods would result in side effects and, therefore, possibly discontinuation. One third of users in this study were using a short-term method for six years, or longer. The Rwandan Family Planning program could improve, and serve women better, if women who need longer periods of protection were encouraged to use long-acting, and even permanent, methods. Use of long-acting, or permanent, methods have the benefit of reducing the time it takes to schedule and travel to providers for method resupply. Additionally, long-term methods are more effective and increase the duration of pregnancy protection. Family planning providers need to emphasize the importance of honoring appointments for resupply not only for pregnancy prevention but also mitigation of side effects.

Variations in the reported experiences of side effects by district are consistent with socio-economic and contraceptive use patterns. Side effects were more frequently mentioned by women in Nyamasheke, a district with lower education levels, higher fertility, and lower modern contraceptive use compared to Musanze. In contrast, in Musanze, family planning users were more likely to identify as not having side effects—even if they did note experiences with side effects in a different part of the interview, and were more likely to be interested in switching for reasons other than experiences of side effects, such as switching from short to long-acting methods. The findings suggest a need for the family planning program in Rwanda to specifically target socio-economically disadvantaged areas with contraceptive information and services in order to sustain or improve further the prevailing levels of contraceptive use.

A strength of the study is the inclusion of the perspectives of both family planning providers, at both the health facility and community levels, as well as experienced modern contraceptive users for triangulation of results to better understand the perspectives of both the providers and the users and to determine where these perspectives converge or diverge. Study participants were drawn from two different districts in Rwanda—purposively selected for having the lowest and highest modern contraceptive prevalence in the country to explore whether experiences around discontinuation and switching differed in the two locales.

This study has a few limitations. Family planning providers recruited women with experience using modern contraceptive methods; it could be that the providers recruited users with positive experiences thereby positively skewing the results. The use of purposive selection of study participants means the results in this study are not generalizable to all Rwandan contraceptive users.

## Conclusion

Both contraceptive users and family planning providers present contraceptive use in Rwanda as an exercise that requires persistence due to experiences of side effects. Once the women made the choice to use contraception, most were committed to consistently using a method to achieve their goals of preventing unintended pregnancy. Family planning providers help users manage side effects if they occur, and if that does not work, advise users to switch methods. Study participants showed, although not always explicitly, an awareness that experiencing side effects was not comparable to the consequences of having unintended births. The participants reported a willingness to persist, and not view discontinuation as an option. The findings suggest that family planning programs can sustain, and increase, contraceptive use levels by training and supporting providers to reach new and dissatisfied users with comprehensive counseling services. Additionally, counseling on side effects at the time of initiating contraceptive use could prepare new users to manage side effects and thus reduce the likelihood of discontinuation.

## Data Availability

The transcripts analyzed for the current study are not publicly available due to the sensitivity of the information, but are available from Western Washington University’s Research Compliance Office, Janai Symons, at lanej4@wwu.edu upon reasonable request.
